# Benefit of rituximab maintenance is associated with Follicular Lymphoma International Prognostic Index in patients with follicular lymphoma

**DOI:** 10.1097/BS9.0000000000000164

**Published:** 2023-08-03

In the article titled “Benefit of rituximab maintenance is associated with Follicular Lymphoma International Prognostic Index in patients with follicular lymphoma,” published on pages 118-124, Volume 5, Issue 2 of *Blood Science*, the figures 2, 3, 4, 5, and 6 have been updated in the online version.
